# New Value to Wool: Innovative Garments for Preservation of Sheep Landraces in Italy

**DOI:** 10.3390/ani11030731

**Published:** 2021-03-08

**Authors:** Ruggiero Sardaro, Piermichele La Sala

**Affiliations:** Department of Economics, University of Foggia, 71121 Foggia, Italy; piermichele.lasala@unifg.it

**Keywords:** animal biodiversity, sheep wool, choice experiments, bass diffusion model, Italy

## Abstract

**Simple Summary:**

Animal landraces are historic local breeds often characterized by low production levels, so that their economic sustainability is often threatened and the risk of extinction is high. In Basilicata, southern Italy, a sheep landrace jeopardized of extinction is Gentile di Puglia. Thus, the study aimed at investigating the feasibility of a possible conservation strategy for such landrace based on the innovative use of its wool for the production of quality garments, so as to give new value to wool and allow further income to farmers. The results highlighted a possible good demand for such products, so as to reduce the difference in gross margin between Gentile di Puglia and the standardized intensively-farmed Comisana, from 57% to 3%. Such economic performance could be further improved by widening the set of fashion wool garments produced, so as to make the Gentile di Puglia even more preferable than other high-production breeds.

**Abstract:**

In Basilicata, southern Italy, a sheep landrace jeopardized of extinction is Gentile di Puglia due to low production levels, low market values of milk and meat, and replacement of wool with synthetic fibers. Due to these dynamics farmers progressively resort to intensive breeding systems, hence causing the gradual disappearance of the ovine sector, the withering of traditional breeding culture and the abandonment of internal and marginal territories. However, in changing climate, traditional agriculture is getting increased attention worldwide by the consumers who are embracing emerging sustainable food production. Thus, in the light of a possible conservation strategy, the study investigates the prospective market for a garment (pullover) produced with wool from Gentile di Puglia, and woven through traditional techniques. An integrated methodological approach based on choice experiments and Bass diffusion model was carried out in order to analyze the consumers’ preferences, the penetration market of this innovative product and the new wool value for farmers. The results pointed out a potential demand focused on women aged 50 years and more and a recognized wool value to farmers of 55 € animal^−1^ (22 € kg^−1^). This new revenue could allow the reduction of the difference in gross margin between the Gentile di Puglia and the non-autochthonous intensively-farmed Comisana from 57% to 3%. The production of further wool garments for a wider demand could increase the economic sustainability of Gentile di Puglia, making it even more preferable than other highly productive breeds.

## 1. Introduction

Important components of agrobiodiversity are landraces, i.e., local varieties of domesticated plant and animal species that have adapted to the natural and cultural local environment. Over the centuries landraces, based on traditional systems of production, ensured food and forage, minimized the production risk, stabilized yields [1], and favored low levels of technology and inputs [2,3,4,5]. Nowadays, landraces contribute to preserve social, cultural, health, recreational and identity values of community, so as to ensure wellbeing [6]. These benefits are mainly supplied in internal and marginal territories, i.e., rural areas characterized by scarce accessibility to main services, such as education, health and mobility. These areas are at risk of depopulation, abandonment, hydrogeological upheaval, and land depletion. However, these areas are also characterized by important natural, human and sociocultural capital that are often considered strategic for their sustainable development [7]. Therefore, landraces have characteristics of public good [8].

Over the last decades, agricultural ecosystems based on local landraces have come increasingly to lose their biological diversity in several areas of the world. Modern intensive cultivation and breeding systems based on few varieties and breeds characterized by higher production levels-but also by higher input levels [9]-have spread in order to increase the global food supply. In recent decades, the focus on production has remarkably changed sheep management, emphasizing the breeding of a small number of very productive and selected cosmopolitan breeds. The risk is the progressive loss of landraces, with negative scenarios in terms of environmental damages and worsening of social wellbeing [10,11]. Thus, the increase of favorable conditions for investments in landraces conservation is desirable, so as to favor the realignment of private interests of farmers with those of community through a regulatory system [12,13,14]. An effective and efficient conservation strategy could be based on the assessment of financial resources aimed at motivating the farmer participation in on-farm conservation programs [15]. In this context, agri-environmental policies (AEPs) can be implemented by funding farmers with financial incentives aimed at enhancing agricultural practices for landraces preservation. In particular, AEPs characterized by an incentive-based structure derive from the Coase Theorem and fall under the Pigouvian framework. They are classified as Payment for Ecosystem Services (PES) [16], aiming to correct market externalities through the implementation of taxes or subsidies. However, another solution could concern the boosting of demand-supply mechanisms focused on products. In this case, preliminary studies concerning the possible market demand of such products are crucial for conservation planning [17].

This study investigates prospective preservation strategies for sheep landraces in Basilicata, southern Italy. Basilicata is the country’s sixth greatest region in the sheep sector, with 248,352 sheep on 3621 farms [18]. The management of these farms is primarily aimed at the production of milk and meat, often registered as Protected Designation of Origin (PDO). In addition, the ovine farms generate positive social and environmental effects in the internal and marginal territories of the region, which are at risk of abandonment due to their environmental and socioeconomic problems (aging population, marginal productive activities, low revenues, lack of infrastructure, hydrogeological instability, etc.). In Basilicata there are landraces (Gentile di Puglia and Leccese) and standardized breeds (Comisana, Sarda, Assaf, Lacaune and Merinizzata), managed through extensive and intensive practices, respectively. However, in recent decades, sheep farming in Basilicata has progressively declined due to reduced meat and milk prices, reduced farm profit, increased production costs, foreign competition and changes in the patterns of consumption. In addition, wool, used for garments, mattresses and cushions, was replaced in part by the spread of synthetic fibers and in part by more competitive foreign wool (Merino wool produced in Australia, China, United States and New Zealand). Thus, nowadays regional wool is processed as a waste, for which farmers do not obtain revenues but pay shearing and disposal costs (3 €/animal). In the period 1990–2010, all these trends caused the 65% and 26% reduction of the sheep and ovine farms, respectively, so as to jeopardize the regional sector [19,20]. These trends more threatened regional landraces, characterized by lower production levels and revenues. In particular, since the mid-1960s, the population of Gentile di Puglia numbered approximately 0.5 million, while in 2013 this landrace counted just 2869 animals in a few dozen farms [21].

In order to preserve the regional sheep landraces, the 2014–2020 Rural Development Programme (RDP) of Basilicata fosters proper strategies to solve the structural and economic problems of the ovine sector by the promotion of skills, processes and management models aimed at the development of innovative products, also in line with the circular economy model [21]. In this context, an innovative use of sheep wool produced by landraces can favor a multifunctional diversification of the regional ovine sector. Important results concern the protection of the regional sheep landraces and related farms, the horizontal aggregation of farmers, the vertical integration of farms so as to create micro supply chains, the growth of new working opportunities and the territorial promotion.

Thus, the study aimed at investigating the feasibility of proper strategies for the development of a wool supply chain in Basilicata based on regional sheep landraces that are able to produce high-quality wool for garments. In particular, the study: (i) estimated the current economic performance of a regional landrace compared to a standardized breed widespread in Basilicata; (ii) analyzed the preferences of potential regional buyers for a textile product made by the landrace wool; (iii) forecasted the market penetration of such wool product among the potential regional buyers; (iv) estimated the landrace wool value to be transferred to regional farmers; (v) analysed the economic performance of the regional landrace in the light of this further wool revenue. Such integrated approach enabled better informed decision making for broader benefit-cost analyses of possible public investment policies.

The paper aims at contributing to the literature by investigating the determinants of consumers’ preferences and the future market demand for the conservation of sheep landraces in general, and in Italy in particular; in addition, it increases the literature on the valuation of the Mediterranean agrobiodiversity components [22,23]. The findings have important implications on the debate about the conservation of Mediterranean animal species and associated costs and benefits; they will also allow checking the suitability of current conservation strategies and designing cost-effective on-farm programs.

## 2. Material and Methods

### 2.1. The Regional Breeds Investigated

The analysis involved a regional landrace, namely Gentile di Puglia, and a non-autochthonous breed, namely Comisana. The origin of Gentile di Puglia can be traced back to ancient Roman times when the soft fleece of an Apulian sheep was used to make the togas of important Roman citizens. There are grounds for suggesting a possible origin of Merino from these ancient Apulian sheep, which spread out from Apulia to North Africa and then to Spain. The name Merino possibly originated from the Mauri, Berber inhabitants of a Roman province called Mauritania [24].

Gentile di Puglia is specialized for wool production. Ewes weigh about 40 kg, the twin lambing birth rate is 20% and, on average, the milk, meat and wool productions are 80–100 kg (per lactation), 25 kg, and 2.50 kg, respectively. Milk contains high levels of protein (6–10%) and fat (8–11%). Usually, flocks contain until 100 animals that are managed through extensive practices based on shifting of pastures over time and feeding mainly with grass and farm hay. The fiber is highly curled, less than 6 cm long, and with a diameter less than 19 μm. Among the regional breeds, these are the best wool characteristics for manufacturing quality garments, therefore the preservation strategy presented in this study focused on Gentile di Puglia. For the aforementioned characteristics, Gentile di Puglia is a landrace due to its following features [25,26,27,28,29,30,31]: (i) its morphological characteristics are distinctive and identifiable; (ii) it is genetically adapted to the conditions of the regional environment; (iii) it did not derive from governmental, organizational, or private breeding programs, and may lack systematic selection, development and improvement by breeders; (iv) it is farmed less deliberately than a standardized breed; (v) it has a historical origin in Apulia and Basilicata regions, has its own local name, and is classified according to intended purpose; (vi) it is characterized by stable yield, even under adverse conditions, but by moderate yield under classic farming conditions; (vii) its genetic heredity shows integrity, but still some heterogeneity (i.e., genetic diversity).

Comisana, instead, is one of the seventeen autochthonous Italian sheep breeds. It is indigenous to central and northern areas of the Mediterranean island of Sicily and it is specialized for milk production. Ewes weigh about 50 kg, the twin lambing birth rate is 65% and, on average, the milk, meat and wool productions amount to 200 kg (per lactation), 40 kg, and 4 kg, respectively. Milk levels of protein (6.5%) and fat (5.2%) are lower than those of Gentile di Puglia. Usually, flocks contain more than 200 animals managed through intensive practices in which pastures are repeated over time, while feeding is mainly based on silage, industrial fodder and concentrates. The fiber is smooth, more than 15 cm long, and with a diameter more than 26 μm. Thus, Comisana wool is not suitable for quality garments but at most for coarse accessories (for example, carpets and blankets).

### 2.2. Gross Margin

The economic performance was assessed through gross margin, which is a profit indicator concerning farm revenue assessed only on management, without considering interest (financial management), taxes (tax management) and depreciation of assets [32].

The survey was based on primary data collected through face-to-face questionnaire-based interviews carried out from May 2019 to January 2020 to regional farmers, and the calculated economic indexes were referred to the accounting year 2018.

Data concerned profit and production costs (mean values) related to 29 Gentile di Puglia farms and 33 Comisana farms, all over the whole region. The questionnaire consisted of four sections, namely: (i) structural (farm area, flock size, sheepfold structure, etc.), (ii) management (nutrition, vaccinations, breed, disease prevention, milking and manure management, etc.), (iii) production (production parameters) and (iv) economic (revenue and costs) characteristics of farms, as well as (v) sociodemographic (gender, age, level of schooling, etc.) characteristics of farmers. Interviews lasted about 40 min, and the accuracy of information provided by each farmer was further checked through on-farm inspections.

### 2.3. Consumer Preferences for Wool Garments

#### 2.3.1. The Consumer Questionnaire

The questionnaire consisted of three sections. The first collected the consumers’ opinions concerning wool garments in general, and the possible use of high-quality garments obtained through regional landrace sheep wool woven by traditional techniques. At the end of Section 1, respondents were informed about the benefits in buying garments obtained from regional sheep landraces in terms of ovine sector preservation and management of the internal and marginal territories. In Section 2, consumers were asked to make choices about a specific type of garment (pullover). In order to check the consistency between hypothetical and real choices [33] and assuming that they are theoretically identical, a supplementary question was inserted at the end of each choice task, where the responses were based on a scale from 0 (very unsure) to 5 (very sure) [34]. In this way, it was possible to account for the risk that respondents attached to each choice, thereby improving the predictive power of the survey [35]. In addition, such question helped to highlight, in the choice task, alternatives that could provide similar utilities as considered substitutes by respondents.

Finally, Section 3 collected sociodemographic characteristics of regional consumers (gender, age, education level, employment, etc.), besides behaviors in buying garments (characteristics of purchase in terms of design, fabric, color and brand; purchase place; etc.). The study area included the ten most populated municipalities of Basilicata (Potenza, Matera, Policoro, Melfi, Pisticci, Lavello, Rionero in Vulture, Lauria, Bernalda and Venosa). Face-to-face interviews lasting approximately 40 min were carried out from July 2019 to March 2020 at shopping centers by four trained economics students at the University of Foggia.

#### 2.3.2. The CE Design

The CE is a stated preference method that allows respondents to express preferences among several alternatives concerning goods, services, projects or programs. Alternatives are defined by different combinations of attributes and respective levels. However, only a few alternatives are selected through an experimental design and grouped so as to define a reasonable number of choice tasks. Finally, for each choice task, respondents are asked to choose the preferred alternative, i.e., the one giving the greatest relative utility [36].

The attributes and the levels were identified by a focus group. In stated preferences studies it ensures design’s quality and content validity to the survey [37] by providing a method for the discussion of concepts and language, for the explanation of scenarios, and for the assessment of the information that respondents require to answer the valuation questions [38]. The focus group meeting was held in Matera in April 2019 and involved two regional farmers, one wool processor, one operator in the artisanal tailoring sector, one operator in the fashion sector, one garment retailer, two final consumers and one regional officer for rural development. These individuals constituted a convenience (non-random) sample for their direct and indirect involvement in the preservation of the regional sheep landraces through the promotion of an innovative wool supply chain. Thus, the participation of a varied and representative sample of stakeholders from different backgrounds was ensured [39]. The participants were invited one month before the meeting via email to provide a general description of the research and the topics of discussion [40]. A recruitment letter containing more specific information on the discussion topics was then distributed to each individual two weeks later [41].

The focus group meeting was designed to last approximately 60 min and was led by a moderator who facilitated and prompted conversation based on the following discussion topics: (i) importance of the regional sheep landraces; (ii) environmental, sociocultural and economic threats for the regional sheep landraces. In particular, a semi-structured interview was used for which three questions were formulated: (1) Are there benefits from farming the regional sheep landraces? (2) Are the regional sheep landraces jeopardized by environmental, social or economic trends? (3) Are there solutions for the preservation of the regional sheep landraces? Finally, as suggested by the literature and practice, the focus group closed with an opportunity for the participants to debrief [42].

The participants recognized several environmental, economic, social and cultural benefits from the regional sheep landraces, and valued as an interesting market solution the valorization of the related wool by promoting the production and the use of specific garments. The attention was focused on one of the most popular wool garments used by people, namely the pullover. Thus, the following attributes and levels were identified (Table 1):

- certified origin of wool for pullover: (i) Extra-EU or EU (no Italy); (ii) Italy (no Basilicata); (iii) Basilicata. For Extra-EU, EU and Italian origin, Merino was considered, while the regional wool was supposed to derive from Gentile di Puglia;
- production area of pullover: (i) Extra-EU or EU (no Italy); (ii) Italy (no Basilicata); (iii) Basilicata. The choice of an Italian or regional product should indicate the interest of consumers in the high-quality sartorial working, in fair work and in the absence of dangerous substances for human health and environment used for breeding and textile processing;
- weaving technique: (i) modern technique: manufacturing of textile products by industrial looms and fully-mechanized processing; (ii) traditional technique: manufacturing of textile products by hand-operated looms, manual processing and knitted trims; it is based on traditional know-how and patterns;
- occasion of use: (i) home: for the usual daily activities; (ii) work: for office, etc.); (iii) special event, for evening with relatives, friends or colleagues, parties, ceremonies, etc.);
- price: the price vector was defined through a market survey on web sites of the main clothing resellers (Amazon, Zalando, AliExpress, Asos, eBay, Privalia, Yoox and Farfetch); in this respect, characteristics and prices of 239 men’s and women’s pullovers for the 2018 autumn/winter season were collected and analyzed.

The color and the time of use (i.e., morning or evening) were not considered among the attributes, since the participants of the focus group did not relate these characteristics to the price. However, preferences for the second characteristics were indirectly caught through the investigation of occasion of use.

An important phase of CE study concerns the experimental design, which allows selection of a suitable number of alternatives. An orthogonal design allowed the selection of 14 profiles, starting from 324 alternatives (2^1^×3^3^×6^1^), besides the “no choice” option. Then 14 choice tasks were assembled and split into two blocks of seven, so that each consumer completed one randomly assigned block (Table 2).

The “no option” was inserted in the choice sets for simulating the mechanism of choice in purchase situations, thus ensuring voluntary nature of participation and conceptual validity of the design. Furthermore, the “pick-one” response format [43] was used for its simulation of real-life decision-making in capturing the first preference, and a 3-alternative design (including the “no option”) was adopted since it seems to generate more participation than a 2-alternative design [44]. The creation of the blocks allowed the reduction of the cognitive effort of the respondents [45], and the alternatives were unlabeled [46] in order to investigate the role of the attributes for the respondents and to increase their attention [47]. Based on this CE design, 500 interviews were planned, 250 for each block. Overall, the study concerned a real and widely used good (woven pullover), therefore, it can be assumed that the risk of biases related to CE was rather low [46].

A quantitative pretesting involving pullover users was carried out, so as to permit the assessment of the potential survey response rate and the item nonresponse rates, as well as the verification of the experimental design’s suitability [48]. The quantitative pre-testing, based on the full version of the questionnaire, was carried out one month before the full survey and involved 37 respondents, who were drawn from the target population at the same shopping centers where the full survey was conducted. The outcomes included a high rate of completed interviews (98.9%), the full comprehension of the questions and proposed scenarios, the absence of any fatigue phenomenon on the part of the respondents, and a successful administration of the questionnaire to individuals with different backgrounds, interests, experiences, and knowledge levels. These findings confirmed that the respondents found the questionnaire and the related decision scenarios comprehensible and credible, thus ensuring a balanced and effective presentation of information [37].

#### 2.3.3. The Econometric Model

The CE theory is based on the concepts of Lancaster [49], according to which the consumers’ utility deriving from purchase is the sum of the utilities related to the characteristics of the good purchased. Therefore, consumers choose a specific good in relation to its specific characteristics. Furthermore, the Random Utility Theory underlying the stochastic utility models [50] states that consumers express their individual preferences in order to maximize utility under the income constraint. Therefore, the choice analysis requests the solution of a utility maximization problem related to a set of alternatives. Considering an individual *n* who chooses the alternative able to ensure the greatest utility among the *J* possible alternatives at a choice opportunity *t*, the utility function is given by the following expression [51]:

(1) $$U_{njt}=V_{njt}+e_{njt},n=1,\ldots ,N;j=1,\ldots ,J;t=1,\ldots ,T$$

where *V_njt_* is the deterministic component, while *e_njt_* is the random component, specifically defined as an independent and identically Gumbel distributed (IID) component. Given a finite set of *J* alternatives, consumer *n* performs a series of pairwise comparisons between them, so as to identify the alternative that maximizes his/her utility. In particular, the alternative *i* will be preferred to *j* if *U_nit_* > *U_njt_*, ∀*j*≠*i*. Due to the stochastic nature of the utility function, the maximization problem can be solved in probabilistic terms. Therefore, considering a set of *J* alternative, the probability that a consumer *n* chooses the alternative *i* is given by:

(2) $$P_{nit}=Prob[(V_{nit}+\varepsilon_{nit})>(V_{njt}+\varepsilon_{njt})]>0,\forall j\neq i,\forall J$$

whose estimation can be made using a discrete choice model [52]. Assuming a linear utility function in parameters for the deterministic component, the expression (1) can be reformulated as:

(3) $$U_{njt}=\beta_{n}x_{njt}+\varepsilon_{njt},n=1,\ldots ,N;j=1,\ldots ,J;t=1,\ldots ,T$$

where *β_n_* is a K × 1 vector of parameters to be estimated, concerns utility, and corresponds to *K* choice characteristics; *x_njt_*, instead, is the K × 1 vector of choice characteristics concerning the alternative *j* at the choice opportunity *t* carried out by the consumer *n*. Consumers may present similar attitudes in presence of different choice sets, leading to correlation phenomena, therefore to the violation of assumption related to the independence of irrelevant alternatives (IIA). In order to relax this assumption, the expression (3) introduces a *β_n_* vector of specific parameters for consumers that follows a *g*(*β*|*θ*) distribution whose vector *θ* indicates mean and variance. This specification allows the formulation of the Random Parameter Logit Model (RPLM), able to capture the heterogeneity of unobserved factors that are common to groups of consumers and are able to influence choices. The probability that a subject *n* chooses the alternative *i* at the choice opportunity *t* is calculated as [49]:

(4) $$P_{nit}=\int \frac{\exp\left(V_{nit}\right)}{\sum_{j}\exp\left(V_{njt}\right)}f\left(\beta \right)d\left(\beta \right)$$

where the distribution *f(·)* of the random parameters *β* is specified by researcher. Since Halton extractions [53] (1960) are an efficient alternative to the random ones [54], the Halton’s method with 1000 extractions was carried out. Furthermore, triangular distribution was used for the functional form of the density functions of parameters [55]. The willingness to pay (WTP) was finally calculated through the ratio between each non-monetary attribute and the attribute relating to price, namely:

(5) $$WTP_{k}=-\frac{\beta_{k}}{\beta_{p}}$$

where *WTP_k_* is the willingness to pay for the *k* attribute, *β_k_* is the estimated coefficient for the *k* attribute and *β_p_* is the estimated coefficient for the price attribute. The 95% confidence intervals were calculated using the method proposed by Krinsky and Robb [56], and the WTP was estimated through the delta method.

### 2.4. Bass Diffusion Model

The manufacturing of a pullover through regional wool processed by traditional weaving techniques is a fashion approach based on innovative technologies, whose adoption often follows an S-shaped curve. Furthermore, cumulated purchases of innovative products can be characterized by three consecutive growth phases: a slow take-up phase, a rapid-growth phase deriving from the knowledge and the spreading of the technology, and a slowing-growth phase when the “not so new” technology tends to saturation.

Diffusion theories try to explain and model the shape of diffusion curves and, consequently, the speed and the shape of cumulative adoption of an innovation among prospective buyers. In marketing, a widely used approach to explain the diffusion of innovations is the Bass model [57]. It allows the forecasting of cumulative sales of a new product, and assumes that potential buyers of an innovation can be divided into two groups:

- innovators, who buy the product first and are influenced only by “external communication”, namely mass media or advertisement;
- imitators, who buy the product if others have already bought it and are influenced by word of mouth or so-called “internal communication”.

The number of first-time purchases *n_t_* at time *t* can be expressed as follows:

(6) $$n_{t}=\frac{dN_{t}}{dt}=p\left(M-N_{t}\right)+q\frac{1}{M}N_{t}\left(M-N_{t}\right)$$

where: *n_t_* are the product purchases in period *t*; *N_t_* are the cumulative product purchases until beginning of period *t*; *M* is the cumulative market potential on the product’s life cycle; *p* is the innovation coefficient; *q* is the imitation coefficient. Integrating over time, the total fraction of the potential that is adopted at time *t* is:

(7) $$F\left(t\right)=\frac{1-e^{-\left(p+q\right)t}}{1+\frac{p}{q}e^{-\left(p+q\right)t}}$$

The above formulas highlight that the diffusion pattern is defined by the estimates of the potential market *M* and by the *p* and *q* coefficients. In this study, the coefficients *M*, *p* and *q* were assessed through CE that, based on a survey questionnaire, allowed to gather information related to sociodemographic and economic characteristics of consumers, as well as on their clothing preferences and purchase behavior. Thus, CE survey was used to assess the potential market *M* of wool pullovers produced through Gentile di Puglia wool and processed by innovative weaving techniques, as well as to classify the respondents as innovators (*p*) and imitators (*q*).

## 3. Results

### 3.1. Farm Economic Performance of Gentile di Puglia in Basilicata

The surveyed farms were very similar to the regional ones as far as the structural characteristics are concerned (Table 3). Conversely, when comparing the farm economic indexes, namely stable gross profit, gross production, specific costs and gross margin, Gentile di Puglia farms were lower than the regional average, while the Comisana farms were higher (Table 4). The modest feeding costs of Gentile di Puglia were due to its rusticity, concerning a greater preference towards wild grass during grazing. In addition, the small veterinary costs were related mostly to the ability to be more resilient to climatic stress and endemic tick-borne parasites [58]. It must outline that these peculiarities, shared by the autochthonous Apulian-Southern breeds, are not embedded in the modern breeds obtained by crossing these native genotypes with Northern ones. Particularly Merinizzata Italiana is a modern breed, created in the first half of the twentieth century or in recent decades by cross-breeding of Gentile di Puglia and Sopravissana stock with imported Merino breeds such as the French Berrichon du Cher and Île-de-France, and the German Merinolandschaf. The aim was to produce a good meat breed without sacrificing wool quality, but unfortunately, without recovering the rusticity. 

Anyhow, due to small yields of milk and meat, the gross margin of Gentile di Puglia is 83% lower than Comisana, in the absence of EU aids, which, conversely, narrow the gap by 26.5%; then, the analysis highlighted a significant difference between intensively and extensively farmed breeds, and, by the way, it pointed out the importance of EU aids in bridging the divide. In any case, EU aids are generally not sufficient to ensure a sufficient economic sustainability in breeding Gentile di Puglia.

### 3.2. Consumer Preferences for Wool Garments

The questionnaires correctly completed were 474. The remaining 26 were excluded as the respondents, all between 18 and 30 years, always chose the “No purchase” option in the choice sets. The characteristics of the respondents were in line with those of regional residents (Table 5). In particular, the persons interviewed were equally distributed between the genders, with a prevalent age between 31 and 50 years, an education level corresponding at most at middle school, mainly employed in the tertiary sector, and characterized by a maximum annual net income of 15,000 euros. The most of respondents used wool pullovers purchased at shopping centers or malls, at the suggestion of friends or of advertisements/promotions on web and media.

The CE analysis showed a good performance of the model, as proved by the Chi-squared and McFadden pseudo-R^2^ (Table 6). Regarding the attributes, the regional wool and the manufacturing area were the most preferred origin characteristics of pullover. These preferences were followed by the Italian (not regional) wool processed in other Italian areas. On the contrary, wool produced and processed in Extra-EU or EU countries (excluding Italy) generated the lowest preferences. Furthermore, traditional weaving techniques were more preferred compared to modern ones, while the use of pullover for special events presented the highest utility, followed by work and home uses. The utility estimates allowed the assessment of the WTP per pullover, split for each attribute and level (Table 7). Thus, the regional origin of wool was appreciated 13.43 € more than the Extra EU and EU (not Italian) wool, while the Italian origin of wool was appreciated 10.69 € less than the regional wool. Concerning the production area, the manufacturing of pullover in Basilicata was appreciated 8.29 € more than the production in Extra EU or EU countries (excluding Italy), while the Italian (not regional) production of pullover was appreciated 2.15 € less than the regional one. For a traditional weaving technique, respondents were willing to pay 13.57 € more than a modern technique, while for a pullover used for special events, they expressed a WTP of 18.02 € more than for a pullover usable at home. The same garment for work use was appreciated 15.26 € less than a pullover for special events.

The analysis of the sociodemographic characteristics of the respondents pointed out specific buyers’ categories and related behaviors towards the choice of the attributes (Table 8). In particular, the WTP tended to increase in case of women, aged between 51 and 65 years, employed in the secondary or tertiary sector, and earning an annual income of over 30.000 €. Gender had no influence in choosing the regional manufacturing area and the use of pullover for work, while the education level influenced positively just the choice of the Italian production area. The annual income affected the choices concerning the production area, the weaving techniques and the use of the garment for special events, while the employment in the secondary sector influenced the choice of the Italian production area, the weaving techniques and the use of pullover for work. Finally, the WTP increased in case of purchase at local markets, shopping centers and malls.

The CE study highlighted the following aspects: (i) the greatest WTP was expressed for pullover made by regional wool, produced in Basilicata, manufactured by traditional weaving techniques, and to use for special events (€ 53.31 more than a pullover with characteristics corresponding to the reference levels of the attributes); (ii) this product was mainly preferred by women, aged more than 50 years, employed in the secondary or tertiary sector, and earning an annual income of 30,000 €; (iii) WTP for the certified regional wool (13.43 € pullover^−1^) can be considered the monetary amount recognized to regional farmers. These findings were used for the following analyses relating the demand and the supply of regional wool.

### 3.3. Diffusion of Wool Garments

Bass model was able to forecast the market penetration of a wool pullover manufactured through regional wool, and processed by innovative weaving techniques. In order to explain and model the shape of diffusion curve, the potential market *M*, as well as the *p* and *q* coefficients, were assessed through information derived from the CE survey. Indeed, the analysis concerning the heterogeneity of CE parameters enabled identification of the potential buyers (*M*) of the most preferred type of pullover. In particular, the wool pullover produced in Basilicata through regional wool and manufactured by innovative weaving techniques was preferred by regional women, aged between 51 and 65 years, employed in the secondary or tertiary sector, and earning an annual income of 30,000 €. There are 281,104 women in Basilicata, of which 64,480 were aged between 51 and 65. Of these 21,393 were working in the secondary or tertiary sector (Istat, 2020) that could be the buyers of the type of pullover proposed here. Thus, this last value indicated the potential market *M* in the Bass model.

Regarding the *p* and *q* coefficients, a question in the CE questionnaire investigated the origin of information that influences the purchase of wool pullovers, so as to classify the respondents as innovators (in case of information got from advertisements and promotions on web and media) or imitators (in case of information from family, friends and acquaintance). Innovators and imitators in the full sample (474) were 31.1% and 63.7%, respectively, while, among the potential buyers of the specific type of pullover defined through the CE were 27.9% and 72.1%. Therefore, the *p* and *q* coefficients used in the Bass model analysis were *p* = 0.279 and *q* = 0.721. Furthermore, a sensitivity analysis based on a ±30% variation of the *p* coefficient was carried out.

The results of Bass model pointed out a good market penetration, among the potential regional buyers, of the specific type of pullover described by CE (Table 9). The innovative product could spread in the regional clothing market within 4–5 years, and results are confirmed in case of a sensible variation of the innovation coefficient *p* (±30%). By considering a wool production per animal of 2.50 kg year^−1^ and a yarn yield of 50%, the present Gentile di Puglia animals in Basilicata (2869) ensure a wool production of 3586 kg year^−1^. Consequently, if the wool used for the production of each pullover varies between 0.3 and 0.6 kg, the potential wool demand for the production of traditional pullovers is between 6418 and 12,836 kg year^−1^, namely between 2 and 4 times higher the present supply. This higher wool request could increase the number of regional Gentile di Puglia animals up to 5134–10,269 units. The estimates confirm the possible success of such strategy for the preservation of Gentile di Puglia in Basilicata.

### 3.4. Farm Economic Performance of Gentile di Puglia in Basilicata by Including Wool Selling

In order to verify the potential performance of a wool supply chain in Basilicata based on the use of Gentile di Puglia wool, a new assessment of the farm gross margin was carried out by including revenues from the selling of greasy Gentile di Puglia wool. To this end, WTP for certified regional wool (13.43 € pullover^−1^) is the value of degreased wool to recognize to regional farmers. Considering a mean degreased wool content per pullover of 0.45 kg, its value was 29.84 € kg^−1^. By subtracting degreasing costs calculated through a specific market survey (8 € kg^−1^), the greasy wool value was 21.84 € kg^−1^. Finally, considering a greasy wool production of 2.50 kg animal^−1^, the greasy wool value was 54.61 € animal^−1^. Thus, starting from the data in Table 4 and adding the new Gentile di Puglia wool revenue, the difference in gross margin between the two considered breeds reduced to 3.2%.

## 4. Discussion and Conclusions

This preliminary study analyzed the potential demand and supply of a specific wool garment in Basilicata, southern Italy, in order to investigate the potential to preserve the Gentile di Puglia sheep in the region. This local and ancient sheep allows low-impact farming techniques that are useful for management of internal and marginal territories. However, the current meat and milk revenues from these sheep are small due to lower production levels. This jeopardizes the survival of this breed in regional flocks. The integrated modelling approach used here demonstrated the potential use of wool for manufacturing garments as a valid solution to economic sustainability of this breed in Basilicata. In particular, the establishment of an innovative supply chain based on the Gentile di Puglia wool could improve returns to farmers and encourage retention of these sheep in preference to alternative breeds. The study focused on one garment and one specific category of customer, and was carried out in Basilicata, a region with low per capita income. The use of this wool also in underwear, accessories, jackets and overcoats and their sale in high income regions of the world to a wider range of consumers could potentially deliver more favorable results in terms of breed preservation and gross margin. Thus, careful marketing strategies could improve profit from local flocks in preference to alternative domestic breeds and even to wool imported from countries with cheaper cost structures. Such a strategy would require investment in collection and sorting of wool, definition of a regional wool brand with quality control and traceability as well as the creation of a supply chain to produce traditional handcrafted textiles from manual processing, through to delivery. Furthermore, such wool application in the textile sector is in line with the Circular Economy Action Plan [59]. Textile products generate the fourth highest pressure for the use of primary raw materials and water, after food, housing and transport, and the fifth highest pressure for GHG emissions. In addition, 60% by value of clothing in the EU is produced in extra EU countries, so that an EU Strategy for Textiles will be defined for strengthening industrial competitiveness and innovation in the sector, as well as for boosting sustainable and circular textiles, textile reuse and new business models. Thus, the approach investigated in this study is fully adherent to the strategies of the Action Plan.

The creation of a wool supply chain for preserving a sheep landrace presents analogies with EU quality schemes, which aims at protecting the names of specific plant and animal products based on their unique characteristics, geographical origin and traditional know-how. In this case, product names are granted with a geographical indication (GI), so as to enable consumers to trust and distinguish quality products, as well as to help producers for better marketing strategies. Italian examples of these sheep products are Pecorino romano, Caciocavallo silano and Canestrato pugliese, while another known not Italian sheep product is the Roquefort cheese from the Lacaune breed. Thus, a similar approach could be carried out for wool from Gentile di Puglia in Basilicata. The breed is related to a complex system of natural and sociocultural capitals in the region based on unique characteristics, geographical origin and traditional know-how. Its preservation goes beyond the productive interests, and ensures the sustainable management of regional internal and marginal territories. However, such economic approach does not exclude other strategies such as out-breeding aimed at improving the genotype of Gentile di Puglia enhancing wool and meat production. Anyway, as already mentioned, crossbreeding implies the loss of the landrace rusticity, which, instead, is crucial to cope up with environmental stress and specific enzootic diseases. In short, as landraces are adapted to the environmental and pedoclimatic conditions of a specific area, it is important to work towards the management of local biodiversity based on the preservation of traditional farming practices; this way will strengthen the fragile land equilibrium by avoiding negative environmental impacts and levering on the boosting of local identities and traditions. Thus, the study of innovative supply chains is crucial to promote Gentile di Puglia and to avoid its extinction.

The production of garments is not the sole strategy for giving new value to Gentile di Puglia wool. Indeed, wool has a mineral composition characterized by high content in K, Na, Fe, and P, thus it has been tried as fertilizer source for plants [60,61,62], binding agent for heavy metals [63], feedstock for composting [64,65] and mulch for weed control [66]. Composted wool has been used as a N source for crop plants such as chickpea and wheat [67,68]. Moreover, wool can be used as insulation material in green building field [69,70]. In particular, sheep wool has interesting characteristics in terms of strength, hydrophobic and hydrophilic properties, thermal performance, ability to regulate temperatures and fire resistance [71].

The characteristics of Gentile di Puglia wool (high curling, small length and reduced diameter) allow its use in fashion, construction and agricultural sectors. Such approach can create new values for farmers, but also for people employed along the respective value chains. Obviously, in reference to the construction and agricultural sectors, further supply chain studies are desirable for decision maker. Anyway, other regional breeds can benefit from the use of their wool in agriculture for a general recovery of the ovine sector. An important step toward all these directions is the enhancement of reuse policy in support of waste prevention and circularity. In addition, the dissemination and implementation of new uses schemes of wool for providing incentives and encouraging sharing of information and good practices are desirable [72,73,74].

## Figures and Tables

**Table 1:** Attributes and their respective levels used in the CE study (The first level is the reference one).

Attributes	Levels
Certified origin of wool	Extra-EU or EU (no Italy) Italy (no Basilicata) Basilicata
Production area of pullover	Extra-EU or EU (no Italy) Italy (no Basilicata) Basilicata
Weaving technique	*Modern* Traditional
Occasion of use	*Home* Work Special event (evening with relatives, friends or colleagues, parties, ceremonies)
Price (€/pullover)	20, 40, 60, 80, 100, 120

**Table 2:** Example of a choice set used for the interviews.

Attributes	Pullover A	Pullover B	No Pullover
Certified origin of wool	Extra-EU or EU (no Italy)	Basilicata	I do not use wool pullover
Production area of pullover	Italy	Basilicata	
Weaving technique	Modern	Traditional	
Occasion of use	Home	Special event	
Price (€/pullover)	60	120	
My choice	☐	☐	☐

**Table 3:** Structural characteristics of survey and regional sheep farms.

Data Source	Ha	0–5	5.1–20	20.1–50	>50	Total-Mean
Survey sample	Animals (n)	510	1588	1910	2427	6435
	Farms (n)	13	29	11	9	62
	Animals/farm	39	55	174	270	134.3
Istat census) (2010)	Animals (n)	21,700	69,606	75,520	96,181	263,007
	Farms (n)	749	1440	702	493	3384
	Animals/farm	29	48	108	195	95.0
Var. % survey sample/Istat census	Animals	−0.3	−1.8	1.0	1.1	
	Farms	−1.2	4.2	−3.0	−0.1	

**Table 4:** Gross margin (€ Animal^−1^) of the surveyed farms (accounting year 2018).

Economic Index	Excluding EU Aids			Including EU Aids			FADN (2018)
	Comisana	Gentile di Puglia	Diff. %	Comisana	Gentile di Puglia	Diff. %	
Meat revenue	11.1	5.6	−49.7	11.1	5.6	−49.7	
Milk revenue	16.9	4.8	−71.5	16.9	4.8	−71.5	
Wool revenue	-	-	-	-	-	-	
Stable gross profit	87.3	28.9	−66.9	87.3	28.9	−66.9	74.6
Reused/Processed production	29.6	10.8	−63.5	29.6	10.8	−63.5	
RDP 2014–2020 (Sub-measure 10.1)	-	-	-	-	27.0	100.0	
Total gross production	144.9	50.1	−65.4	144.9	77.1	−46.8	102.8
Lamb feeding cost	4.3	3.6	−16.3	4.3	3.6	−16.3	
Fodder cost	20.3	15.6	−23.2	20.3	15.6	−23.2	
Concentrates cost	5.5	2.4	−56.4	5.5	2.4	−56.4	
Veterinary and sanitation cost	9.1	7.8	−14.3	9.1	7.8	−14.3	
Watering cost	0.8	0.7	−12.5	0.8	0.7	−12.5	
Shearing and disposal cost	3.0	3.0	0.0	3.0	3.0	0.0	
Total specific costs	43.0	33.1	−23.0	43.0	33.1	−23.0	39.0
Gross margin	101.9	17.0	−83.3	101.9	44.0	−56.8	61.9

**Table 5:** Sociodemographic characteristics of the regional sample.

Variables	Categories/Ranges	Sample Respondents	Sample Respondents	Regional Census 2020
		(n.)	(%)	(%)
Gender	Male	244	48.8	49.2
	Female	256	51.2	50.8
Age (years)	18–30	87	17.4	15.5
	31–50	176	35.2	34.1
	51–65	142	28.4	30.0
	65–79	95	19.0	20.4
Education level	None/ Primary school/Middle school	327	65.4	62.9
	Secondary school/Bachelor degree	173	34.6	37.1 ^a^
Employment	Primary sector	52	10.4	8.2
	Secondary sector	143	28.6	26.7
	Tertiary sector	305	61.0	65.1 ^a^
Net income (€ year^−1^)	0–15,000	295	59.0	54.8 ^a^
	15,001–30,000	153	30.5	32.5
	>30,000	53	10.5	12.7
Do you use wool pullovers (wool >30%)?	Yes	474	94.8	
	No	26	5.2	
Usual place of purchase of wool pullovers	Local market	145	30.5	
	Shopping center, Mall	204	43.1	
	Atelier	82	17.4	
	Boutique	43	9.0	
Origin of information influencing the purchase of wool pullovers	Family	44	9.3	
	Friends	147	31.1	
	Acquaintance	127	26.8	
	Advertisements/promotions from web and media	155	32.8	

^a^ The superscript letter indicates a significant difference (*p* < 0.05) between the respondents of the sample and the reference population (the residents of Basilicata region), based on the *t*-test analysis.

**Table 6:** Utility estimates for the characteristics of wool pullover.

Choice	Coefficient	Std. Err.	
Random parameters in utility functions			
Certified origin of wool: Italy (no Basilicata)	0.115	0.048	^**^
Certified origin of wool: Basilicata	0.564	0.181	^***^
Production area of pullover: Italy (no Basilicata)	0.258	0.101	^**^
Production area of pullover: Basilicata	0.348	0.133	^**^
Weaving technique: Traditional	0.570	0.144	^***^
Occasion of use: Work	0.116	0.050	^**^
Occasion of use: Special event	0.757	0.141	^***^
Nonrandom parameters in utility functions			
Price	−0.042	0.010	^***^
ASC	−3.113	0.729	^***^
Standard deviations of parameter distributions			
Certified origin of wool: Italy (no Basilicata)	2.132	0.517	^***^
Certified origin of wool: Basilicata	1.755	0.331	^***^
Production area of pullover: Italy (no Basilicata)	4.172	1.059	^***^
Production area of pullover: Basilicata	2.290	0.549	^***^
Weaving technique: Traditional	5.283	0.980	^***^
Occasion of use: Work	3.176	0.773	^***^
Occasion of use: Special event	6.279	1.764	^***^
Obs.: 3318			
Log likelihood function: −5211.28			
Chi-squared: 5025.36			
Significance level: <0.0001			
McFadden pseudo-R^2^: 0.3531			

^***^: sign. 1%; ^**^: sign. 5%.

**Table 7:** Willingness to Pay for the characteristics of wool pullover.

Items	Levels	WTP		C.I. 95%	
(a)	Certified origin of wool: Italy (no Basilicata)	2.74	^***^	1.93	3.55
(b)	Certified origin of wool: Basilicata	13.43	^***^	10.55	16.30
(c)	Production area of pullover: Italy (no Basilicata)	6.14	^***^	4.47	7.82
(d)	Production area of pullover: Basilicata	8.29	^***^	5.97	10.60
(e)	Weaving technique: Traditional	13.57	^***^	10.94	16.20
(f)	Occasion of use: Work	2.76	^**^	1.91	3.61
(g)	Occasion of use: Special event	18.02	^***^	15.01	21.03
Traditional pullover by Gentile di Puglia wool (b+d+e+g)		53.31	^***^	42.49	64.13

^***^: sign. 1%; ^**^: sign. 5%.

**Table 8:** Socioeconomic characteristics influencing choice for wool pullover.

**Characteristics**	**Certified Origin of Wool: Italy (No Basilicata)**			**Certified Origin of Wool: Basilicata**			**Production Area of Pullover: Italy (No Basilicata)**			**Production Area of Pullover: Basilicata**		
Female	0.771	0.346	^**^	0.746	0.289	^**^	0.829	0.144	^***^	0.580	0.457	
18–30 years	0.673	1.923		0.202	0.182		0.352	0.251		0.275	0.166	
51–65 years	0.725	0.273	^**^	0.484	0.205	^**^	0.911	0.410	^**^	0.548	0.220	^**^
Sec. school-Bach. degr.	0.341	0.216		0.251	0.176		0.495	0.212	^**^	0.536	0.350	
Secondary sector	0.274	0.217		0.673	0.510		0.583	0.218	^**^	0.114	0.070	
Tertiary sector	0.286	0.075	^***^	0.542	0.130	^***^	0.668	0.270	^**^	0.265	0.061	^***^
>30,000 €	0.572	0.381		0.855	0.515		0.502	0.155	^***^	0.639	0.246	^**^
Loc. mark./Shopp. center/Mall	−0.683	0.256	^**^	−0.734	0.195	^***^	−0.310	0.124	^**^	−0.215	0.078	^**^
	**Weaving Technique: traditional**			**Occasion of Use: Work**			**Occasion of Use: Special Event**			**Price**		
Female	0.475	0.070	^***^	−0.371	0.275		0.661	0.181	^***^	0.685	0.247	^**^
18–30 years	−0.816	0.850		0.428	0.404		0.261	0.168		0.472	0.306	
51–65 years	0.495	0.095	^***^	0.515	0.157	^***^	0.575	0.107	^***^	0.639	0.137	^***^
Sec. school-Bach. degr.	0.163	0.098		0.208	0.136		0.173	0.149		0.811	0.567	
Secondary sector	0.287	0.121	^**^	0.162	0.060	^**^	0.202	0.155		0.607	0.690	
Tertiary sector	0.925	0.201	^***^	0.411	0.170	^**^	0.429	0.158	^**^	0.892	0.192	^***^
>30,000 €	1.701	0.322	^***^	0.269	0.198		0.527	0.208	^**^	1.330	0.251	^***^
Loc. mark./Shopp. center/Mall	−0.944	0.169	^***^	0.167	0.097		−0.326	0.091	^***^	−1.507	0.320	^***^

^***^: sign. 1%; ^**^: sign. 5%.

**Table 9:** Estimates of the Bass diffusion model.

Year	M = 21,393; *p* = 0.195; *q* = 0.805				M = 21,393; *p* = 0.279; *q* = 0.721				M = 21,393; *p* = 0.363; *q* = 0.637			
t	Sales Forecast. (n)	Cum. Sales Forecast. (n)	Sales Forecast. (%)	Cum. Sales Forecast. (%)	Sales Forecast. (n)	Cum. Sales Forecast. (n)	Sales Forecast. (%)	Cum. Sales Forecast. (%)	Sales Forecast. (n)	Cum. Sales Forecast. (n)	Sales Forecast. (%)	Cum. Sales Forecast. (%)
1	4172	4172	0.20	0.20	5969	5969	0.28	0.28	7766	7766	0.36	0.36
2	6061	10,233	0.28	0.48	7406	13,375	0.35	0.63	8098	15,863	0.38	0.74
3	6473	16,707	0.30	0.78	5851	19,226	0.27	0.90	4619	20,483	0.22	0.96
4	3860	20,567	0.18	0.96	2009	21,235	0.09	0.99	886	21,368	0.04	1.00
5	801	21,367	0.04	1.00	157	21,392	0.01	1.00	25	21,393	0.00	1.00
6	26	21,393	0.00	1.00	1	21,393	0.00	1.00	0	21,393	0.00	1.00
7	0	21,393	0.00	1.00	0	21,393	0.00	1.00	0	21,393	0.00	1.00
8	0	21,393	0.00	1.00	0	21,393	0.00	1.00	0	21,393	0.00	1.00
9	0	21,393	0.00	1.00	0	21,393	0.00	1.00	0	21,393	0.00	1.00
10	0	21,393	0.00	1.00	0	21,393	0.00	1.00	0	21,393	0.00	1.00

## Data Availability

Data will be available on request due to privacy restrictions.
